# Prevalence of antimicrobial-resistant organisms in smaller Canadian hospitals: Community, Rural, and Northern Acute Care Point Prevalence (CNAPP-19) Survey, 2019

**DOI:** 10.14745/ccdr.v48i1112a09

**Published:** 2022-11-03

**Authors:** Shari Thomas, Denise Gravel Tropper, Braden Knight, Donald Sheppard, Tanya Lary, Jami Mackenzie, Greg German, Charles Frenette, Kathryn Bush, Jennifer Ellison, Jennifer Happe, Jayson Shurgold

**Affiliations:** 1 Antimicrobial Resistance Task Force, Public Health Agency of Canada, Ottawa, ON; 2 Unity Health, Toronto, ON; 3 McGill University Health Centre, Montréal, QC; 4 Alberta Health Services, Calgary, AB; 5 Infection Prevention and Control Canada, Calgary, AB

**Keywords:** point prevalence study, antimicrobial resistance, antimicrobial resistant organisms, *Clostridioides difficile* infection, methicillin-resistant *Staphylococcus aureus*, vancomycin-resistant *Enterococci*, carbapenemase-producing Enterobacterales, *Escherichia coli*, nosocomial infections

## Abstract

**Background:**

The availability of national data on the prevalence of antimicrobial resistant infections in smaller, community, northern and rural acute care hospitals is limited. The objective of this article is to determine the prevalence of infections caused by selected antimicrobial-resistant organisms (AROs) in these smaller hospitals.

**Methods:**

A point prevalence survey was conducted by 55 hospitals between February and May 2019 and included representation from all 10 Canadian provinces. Eligible hospitals were those with 350 or fewer beds. Data were collected on hospital characteristics. De-identified patient data were collected on selected infections (pneumonia, urinary tract infections, bloodstream infections, skin/soft tissue infections, surgical site infections, and *Clostridioides difficile* infections) for selected AROs (methicillin-resistant *Staphylococcus aureus*, vancomycin-resistant *Enterococci*, extended-spectrum β-lactamase–producing organisms and carbapenemase-producing organisms). Data on antimicrobial prescribing and infection prevention and control precautions were also collected.

**Results:**

A total of 3,640 patients were included in the survey. Median patient age was 73 years, and 52.8% (n=1,925) were female. Selected infections were reported in 14.4% (n=524) of patients, of which 6.9% (n=36) were associated with an ARO infection. Infection prevention and control additional precautions were in place for 13.7% (n=500) of patients, of which half (51.0%, n=255) were due to an ARO. Approximately one third (35.2%, n=1,281) of patients had at least one antimicrobial prescribed.

**Conclusion:**

Antimicrobial-resistant organisms remain a serious threat to public health in Canada. The results of this survey warrant further investigation into AROs in smaller Canadian hospitals as a potential reservoir of antimicrobial resistance.

## Introduction

Antimicrobial resistance (AMR) is a serious threat to public health, as it erodes the efficacy of commonly used therapies in treating and preventing a wide range of infectious diseases ((1)). Infections by antimicrobial-resistant organisms (ARO) are associated with increased hospitalization costs, greater disease severity, and poor patient outcomes ((2)).

Surveillance is a key component to support efforts to reduce the burden of illness associated with AROs. The Canadian Nosocomial Infection Surveillance Program (CNISP) has prospectively monitored healthcare-associated infections (HAI) in larger tertiary care hospitals in major urban areas ((3,4)), including a subset of infections caused by AROs that have been prioritized by the Public Health Agency of Canada (PHAC) ((5)). Data on AMR in smaller, non-academic hospitals (often located in community, rural and northern regions) remain limited ((3)). The Community, Rural, and Northern Acute Care Point Prevalence (CNAPP) survey, administered by PHAC, was designed to assess the burden of AMR and antimicrobial use (AMU) in this underrepresented area of the Canadian healthcare system.

The primary study objective was to describe the prevalence of selected infections in participating hospitals on the date of the point-prevalence survey. Secondary objectives were to describe the prevalence of AMU, screening practices related to AROs and the prevalence of patients under additional infection prevention and control (IPAC) precautions.

## Methods

### Survey design and sampling

This study was an observational point prevalence study conducted by PHAC. Information was collected on hospital characteristics and de-identified patient information through two respective standardized questionnaires ((6)), one at the hospital level and one at the patient level. The CNAPP survey was adapted from existing CNISP point prevalence surveys and materials ((4)). Eligible hospitals were those with fewer than 350 acute care beds. Hospitals that provided only day and overnight surgery, rehabilitation, psychiatric care, paediatric care, palliative care, outpatient clinics, maternity services or long-term care were ineligible to participate. Sites that provided these services in addition to other eligible services were included; however, patients from those ineligible areas were excluded from the hospital census for the purpose of CNAPP. Hospital sites were recruited by convenience sampling using pre-existing professional associations and relationships; efforts were made to recruit representation from all Canadian provinces. Data were collected by nurses, pharmacists, IPAC staff, or infectious disease physicians (based on facility specific availability). Training was provided to all participating sites. The survey was conducted during a 24-hour period between February 1, 2019, and March 30, 2019 (except hospitals in Québec, which conducted the survey between April 1, 2019, and May 31, 2019).

The hospital questionnaire consisted of twelve questions relating to the size and services of the facility, hospital screening practices and antimicrobial stewardship practises (**Supplemental material S1**). Data pertaining to the hospital (hospital questionnaire) and eligible patients (patient questionnaire) were obtained from patient hospital charts, nurses’ logs, laboratory reports and administrative systems, or by any other means as seen appropriate by the participating hospital.

The patient questionnaire consisted of eight questions relating to patient demographics, additional IPAC precautions, presence of selected infections (pneumonia, urinary tract infections [UTI], bloodstream infections [BSI], skin/soft tissue infections [SSTI], surgical site infections [SSI] and *Clostridioides difficile* infections [CDI]), presence of selected AROs and antimicrobials prescribed (**Supplemental material S2**).

### Setting and participants

All inpatients in acute care units were identified using the hospital census. Patient information was collected over one 24-hour period, starting at 8:00 a.m. on the date of the hospital census and ending at 8:00 a.m. the following day. Data were collected retrospectively to ensure that all patient charts were updated with eligible information (e.g. swabs taken on the date of the survey). The survey collected patient-level data on demographics, transmission-based precautions, presence of specific infections, presence of selected AROs and antimicrobial use. Selected infection types included: pneumonia, UTIs, BSIs, SSTIs, SSIs and CDIs. Definitions for selected infections can be found in **Appendix A1**. An infection was considered to be present if a patient was symptomatic or receiving antimicrobial therapy for the treatment of the infection at the time of the hospital census. As the census day elapses 24 hours (from 8:00 a.m. to 8:00 a.m.), isolates recovered prior to 8:00 a.m. on the day following the census were eligible to be included in the prevalence survey.

The AROs selected for inclusion in the survey were aligned to PHAC priority organisms ((5)), and included methicillin-resistant *Staphylococcus aureus* (MRSA), vancomycin-resistant *Enterococci* (VRE), extended-spectrum β-lactamase (ESBL)–producing organisms and carbapenemase-producing organisms CPOs. Definitions used in this point-prevalence survey, including those for selected AROs, are the same as those used by CNISP. Detailed case definitions can be found in **Appendix A1**.

This prevalence survey was observational and did not involve any alteration to patient routine care. As such, this study was considered exempt from the requirement for ethics approval as a quality assurance study within the mandate of hospital infection prevention and control programs or approved by the research and ethics boards at participating hospitals if required by institution-specific policies. A unique encrypted identifier linked to patient name was used to identify patients at the participating hospitals and was not disclosed to PHAC. All data were strictly confidential.

### Data analysis

We described the characteristics of participating hospitals and patients that were surveyed, the prevalence of selected infections and selected AROs and AMU. We compared the characteristics of patients with selected infections to those who did not have selected infections, using chi square tests to calculate *p*-values. A bivariate analysis of selected infections and AROs was performed to assess the prevalence of AROs contributing to these infections. Prevalence was calculated as the proportion of patients with an infection/ARO divided by the total population, multiplied by 100. Mean hospital prevalence was calculated as the mean of each individual hospital’s prevalence for each infection/ARO; 95% confidence intervals (CI) were calculated for all means and proportions. Data analysis was conducted in Microsoft Excel and SAS EG 7.1 (Cary, North Carolina).

## Results

### Hospitals

A total of 55 hospitals from 10 provinces with a combined total of 4,159 beds participated in the survey between February 6, 2019, and May 21, 2019. Hospitals in two territories expressed interest in participating but were unable to at the time of the study. Median hospital size was 53 beds (n=5 to 347 beds). While all Canadian provinces were represented in the study, participation varied by province. Facilities in Eastern Canada were, on average, smaller than hospitals in Western and Central Canada. All surveyed hospitals provided medical services, and none provided services for solid organ transplant, bone marrow transplant, paediatric intensive care or burn care. **Table 1** further describes the characteristics of the hospitals that participated in the survey.

**Table 1:** Characteristics of participating hospitals (n=55)

Variable	N	%
Provincial distribution		
BC	5	9.1
AB	8	14.6
SK	3	5.5
MB	6	10.9
ON	7	12.7
QC	9	16.4
NB	2	3.6
NS	9	16.4
PE	2	3.6
NL	4	7.3
Regional distribution		
Eastern	17	30.9
Central	16	29.1
Western	22	40.0
Hospital size distribution (number of beds)		
Median	53	N/A
Mean	76	N/A
Range	5–347	N/A
Distribution by availability of services in each facility^a^		
Medical	55	100
Surgical	42	76.4
Obstetrics & gynecology	37	67.3
Paediatric	30	54.6
Dialysis	25	45.5
Rehabilitation	19	34.6
Other^b^	19	34.6
Oncology	18	32.7
LTC	17	30.9
Trauma	12	21.8
ICU, neonatal	7	12.7
Solid organ transplant	0	0
Bone marrow transplant	0	0
Burn unit	0	0
Screening at admission		
MRSA	55	100
VRE	43	78.2
CPO	39	70.9
ESBL	5	9.1
Screening after admission^c^		
MRSA	48	87.3
VRE	39	70.9
CPO	38	69.1
ESBL	9	16.4
Hospitals with at least one selected ARO infection	25	45.4
MRSA	14	25.5
VRE	2	3.6
ESBL	11	20.0
CPO	0	0

Abbreviations: AB, Alberta; ARO, antimicrobial-resistant organism; BC, British Columbia; CPO, carbapenemase-producing organisms; ESBL, extended-spectrum β-lactamase producing organisms; ICU, intensive care unit; LTC, long-term care; MB, Manitoba; MRSA, methicillin-resistant *Staphylococcus aureus*; N/A, not applicable; NB, New Brunswick; NL, Newfoundland and Labrador; NS, Nova Scotia; ON, Ontario; PE, Prince Edward Island; QC, Québec; SK, Saskatchewan; VRE, vancomycin-resistant *Enterococci*

^a^ Services available at the facility. As described in the methods, not all services were included in the Community, Rural, and Northern Acute Care Point Prevalence survey

^b^ Other includes special care units, psychiatric units, mental health and addictions care, etc.

^c^ Includes screening of close contacts of new cases, periodic ward surveys and/or targeted units only

Antimicrobial-resistant organism screening practices at admission varied by hospital (e.g. screening all patients as part of admission, screening patients based on risk criteria or only screening patients admitted to medical and surgical wards). All centres performed some screening for MRSA at admission, 78.2% (n=43) for VRE, 70.9% (n=39) for CPOs and only 9.1% (n=5) for ESBL-producing organisms. The ARO screening practices after admission also varied (e.g. screening close contacts of new cases, periodic ward surveys, screening of targeted units). More than two thirds of the participating hospitals screened some patients for MRSA (n=48), VRE (n=39) or CPO (n=38) after admission; however, fewer than one in five (n=9) hospitals screened for ESBL-producing organisms at any point after admission. The ESBL-producing organisms were the only selected ARO for which more hospitals screened patients during their stay rather than upon admission (Table 1).

At least one patient with an MRSA infection was reported from 14 hospitals (25.5%) and patients with ESBL-producing organisms were reported from 11 hospitals (20.0%). Only two hospitals (3.6%) reported VRE infections and no hospitals reported patients with CPO infection.

### Patients

A total of 3,640 patients were identified from hospital census during a 24-hour period between February 6, 2019, and May 21, 2019 (inclusive). A slight majority (52.8%) of those included were female and one third of patients were 65 years of age or older (66.4%). The median patient age was 73 years old, ranging from newborns to 103 years of age. The geographic distribution was similar to that of hospitals, in that the largest proportion came from Western Canada (43.6%). Almost half of patients (47.7%) were located in a medical ward; 19.5% were in a surgical ward and 12.4% were in a mixed medical/surgical ward. **Table 2** further describes the characteristics of the patients that were included in the survey.

**Table 2:** Patient characteristics (n=3,640)

Characteristics		With selected infections (n=524)	%	Without selected infection (n=3,116)	%	*p*-value	Total population (N=3,640)	%
Region *p*=0.02	Eastern	109	20.80	686	22.02	N/A	795	21.84
	Central	209	39.89	1,048	33.63	N/A	1,257	34.53
	Western	206	39.31	1,382	44.35	N/A	1,588	43.63
Sex *p*=0.81	Male	250	47.71	1,465	47.02	N/A	1,715	47.12
	Female	274	52.29	1,649	52.92	N/A	1,923	52.83
	Other	0	0.0	2	0.06	N/A	2	0.05
Age *p*=0.03	Mean (SD)	67.43 (20.36)	N/A	67.76 (21.69)	N/A	0.75	67.7 years (21.50)	N/A
	Median	72	N/A	73	N/A	N/A	73 years	N/A
	Infants (<1 year)	4	0.76	82	2.63	N/A	86	2.36
	Children (1–17 years)	9	1.72	52	1.67	N/A	61	1.68
	Adults (18–64 years)	172	32.82	903	28.98	N/A	1,075	29.53
	Seniors (>65 years)	339	64.69	2,079	66.72	N/A	2,418	66.43
Location of patient on survey day *p*<0.01	Medical	247	47.14	1,488	47.75	N/A	1,735	47.66
	Surgical	105	20.04	607	19.48	N/A	712	19.56
	Mixed medical/surgical	58	11.07	393	12.61	N/A	451	12.39
	ICU	31	5.92	154	4.94	N/A	185	5.08
	Adult ICU	31	5.92	99	3.18	N/A	130	3.57
	Neonatal ICU	0	0.0	55	1.77	N/A	55	1.51
	Mixed ICU/CCU	0	0.0	34	1.09	N/A	41	1.13
	Hematology/oncology/bone marrow transplant	15	2.86	40	1.28	N/A	55	1.51
	Paediatrics	13	2.48	71	2.28	N/A	84	2.31
	Coronary care	1	0.19	26	0.83	N/A	27	0.74
	Obstetrics	2	0.38	83	2.66	N/A	85	2.34
	ER	32	6.11	144	4.62	N/A	176	4.84
	Step down unit	4	0.76	12	0.39	N/A	16	0.44
	Other	9	1.72	64	2.05	N/A	73	2.01
Patients prescribed antimicrobials	At least one antimicrobial	505	96.37	776	24.90	<0.01	1,281	35.19
	Multiple antimicrobials	195	37.21	232	7.45	<0.01	427	11.73
Patients on additional IPAC precautions	For any reason	140	26.72	360	11.55	<0.01	500	13.7
	Due to selected ARO	65	12.40	190	6.10	<0.01	255	7.01

Abbreviations: ARO, antimicrobial-resistant organism; CCU, critical care unit; ER, emergency room; ICU, intensive care unit; IPAC, infection prevention and control; N/A, not applicable; SD, standard deviation

One in seven patients (14.4%) had at least one selected infection (n=524). Of these, 27.8% (n=146) were healthcare-associated (4.0% of all patients). Urinary tract infections and pneumonia were the most commonly reported infections (each of them accounting for almost 4.1 per 100 inpatients; 95% CI, 3.4–4.7), while SSI were the least commonly reported (0.8 per 100 inpatients; 95% CI, 0.5–1.1). Considering hospital size, the mean hospital prevalence of selected infections followed a similar distribution to the aforementioned distribution of overall prevalence, with pneumonia having the highest mean hospital prevalence (4.6; 95% CI, 2.9–6.2), followed by UTIs (4.3; 95% CI, 3.2–5.3) and SSTIs (3.1; 95% CI, 2.3–3.9). The SSIs had the lowest mean hospital prevalence (0.7; 95% CI, 0.4–0.9) (**Table 3**).

**Table 3:** Mean prevalence of selected antimicrobial resistant organisms and selected infections

Selected infections	N	Proportion of patients (per 100 inpatients)		Mean hospital prevalence	
		n	95% CI	n	95% CI
Patients with selected infections					
UTI	149	4.09	3.45, 4.74	4.26	3.20, 5.32
Pneumonia	148	4.07	3.42, 4.71	4.56	2.93, 6.19
SSTI	112	3.08	2.52, 3.64	3.09	2.27, 3.90
BSI	90	2.47	1.97, 2.98	1.67	1.12, 2.23
CDI	34	0.93	0.62, 1.25	1.44	0.0, 3.27
SSI	30	0.82	0.53, 1.12	0.65	0.37, 0.93
Patients with selected ARO infections					
MRSA	18	0.49	0.27, 0.72	0.44	0.19, 0.69
VRE	4	0.11	0.0, 0.22	0.04	0.0, 0.11
ESBL	14	0.38	0.18, 0.59	0.25	0.09, 0.41
CPO	0	0	0	0	0

Abbreviations: ARO, antimicrobial-resistant organism; BSI, bloodstream infection; CDI, *Clostridioides difficile* infection; CI, confidence interval; CPO, carbapenemase-producing organisms; ESBL, extended-spectrum β-lactamase producing organisms; MRSA, methicillin-resistant *Staphylococcus aureus*; SSI, surgical site infection; SSTI, skin/soft tissue infection; UTI, urinary tract infection; VRE, vancomycin-resistant *Enterococci*

The characteristics of patients with selected infections were like those who did not have selected infections, except that patients with selected infections were more likely to be prescribed antimicrobials than those who did not have selected infections (96.4% of patients with selected infections compared to 24.9% of patients without selected infections *p*<0.01) (Table 2).

In total, we identified 36 patients with 39 unique infections from which a selected ARO was recovered, for a prevalence of 1.0% of the total patient population (n=36/3,640) and 6.9% of patients with a selected infection (n=36/524). Almost twice as many females as males were affected by these ARO infections. Eighteen patients were infected with MRSA (0.5 per 100 inpatients; 95% CI, 0.3–0.7); of these 18 patients, three were infected at multiple sites, 14 were infected with an ESBL-producing organism (0.4 per 100 inpatients; 95% CI, 0.2–0.6) and four were infected with VRE (0.1 per 100 inpatients; 95% CI, 0.0–0.2). One of the patients infected with VRE had concurrent CDI. No patients were reported to have CPO infections (Table 3).

Five hundred patients were under additional infection prevention and control precautions (13.7% of total patients). Of these 500 patients, 255 (51.0%) were under additional precautions due to an ARO. Patients with a selected infection were more likely to be on additional precautions than those who did not have a selected infection (26.7% compared to 11.6%, respectively, *p*<0.01). This was also true of patients who were on additional precautions due to an ARO (12.4% compared to 6.1%, respectively, *p*<0.01) (Table 2). The most common additional precautions were contact (n=468, 93.6% of patients on additional precautions), followed by droplet (n=157, 31.4%), cohorting (n=9, 1.4%), airborne and other (both n=7, 1.4%). Other precautions encompassed those patients who were placed on additional precautions due to their length of stay or other facility specific policies.

Among all selected infections caused by an ARO, BSIs were most frequent (11.1%; 95% CI, 4.6–17.6), followed by SSTIs (8.9%; 95% CI, 3.6–14.2) and UTIs (8.7% of UTIs; 95% CI, 4.2–13.3) (**Table 4**).

**Table 4:** Selected antimicrobial resistant organisms by selected infection type^a^

Infection type	Total patients with selected infection	MRSA	VRE	ESBL-producing organisms	CPO	Selected infections caused by (one or more) selected AROs	Selected infections caused by (one or more) selected AROs	
	n	n	n	n	n	n	%	95% CI
UTI	149	1	1	11	0	13	8.7%	4.2–13.3
Pneumonia	148	3	0	1	0	4	2.7%	0.1–5.3
SSTI	112	10	0	0	0	10	8.9%	3.6–14.2
BSI	90	5	3	2	0	10	11.1%	4.6–17.6
SSI	30	2	0	0	0	2	6.7%	0–15.6
CDI	34	N/A	N/A	N/A	N/A	N/A	N/A	N/A

Abbreviations: ARO, antimicrobial-resistant organism; BSI, bloodstream infection; CDI, *Clostridioides difficile* infection; CI, confidence interval; CPO, carbapenemase-producing organisms; ESBL, extended-spectrum β-lactamase producing organisms; MRSA, methicillin-resistant *Staphylococcus aureus*; N/A, not applicable; SSI, surgical site infection; SSTI, skin/soft tissue infection; UTI, urinary tract infection; VRE, vancomycin-resistant *Enterococci*

^a^ Infection sites were not mutually exclusive (i.e. patients could have multiple selected infections associated with multiple selected AROs): 36 patients had infections with selected AROs, with three patients having AROs in multiple sites (two patients with MRSA BSI and MRSA pneumonia one patient with MRSA BSI and MRSA SSTI)

### Antimicrobial use

**Figure 1:**
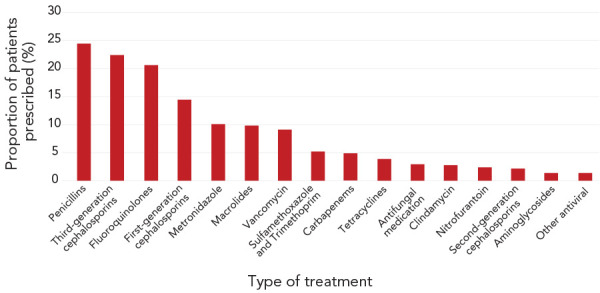
Prevalence of treatments^a,b^ among patients surveyed (n=1,281) ^a^ Treatment categories are not mutually exclusive (i.e. patients can be prescribed more than one antimicrobial) ^b^ Other antibiotics, daptomycin, linezolid, other non-antimicrobials, anti-tb medication, aztreonam and colostin were all prescribed for <1% of patients

On the day of the census, 35.2% (95% CI, 33.6–36.7) of patients were being prescribed at least one antimicrobial and 11.7% of patients were being prescribed more than one antimicrobial. Antimicrobial use was most prevalent among the oldest patients. Among patients of all ages who received an antimicrobial, penicillin-class antibiotics were the most prevalent prescriptions (24.4%), followed by third-generation cephalosporins (22.4%), fluoroquinolones (20.6%), first-generation cephalosporins (14.4%), metronidazole (10.1%), macrolides (9.8%) and vancomycin (9.1%). **Figure 1** further describes the prevalence of antimicrobial use in the study population.

More than half (60.8%) of AMU was prescribed empirically (without microbiologic laboratory results), compared to 22.8% prescribed as targeted therapy (accompanied by microbiologic laboratory results) and 11.9% as prophylactic therapy. The reason for prescription was unknown for 4.8% of prescriptions.

Among patients with an ARO infection (n=36), penicillins were the most commonly prescribed antimicrobial class (27.8%), followed by carbapenems (19.4%), fluoroquinolones (16.7%), first generation cephalosporins (11.1%) and third-generation cephalosporins (8.3%).

## Discussion

We measured the burden of specific infections and selected AROs among small, community hospitals in Canada based on findings from a point prevalence survey administered in 2019. The overall prevalence of infections in our survey was14.4%, while the prevalence of HAIs was 4.0%. This is similar to what has been reported from large tertiary care hospitals by the United States Centers for Disease Control and Prevention (4.0% in 2011 and 3.2% in 2015) ((7)), and lower than reported from the European Centre for Disease Control and Prevention (7.1% in 2016/2017) ((8)) and previous CNISP point prevalence surveys (11.3% in 2009 and 7.9% in 2017) ((9)). Our study showed a CDI prevalence of 0.9 per 100 inpatients. This is consistent with other studies from large Canadian hospitals as well as from hospitals in many other countries ((5,10,11)). Pneumonia and UTI were the most prominent selected infections in our study. This is similar to what has been reported by point prevalence surveys in larger Canadian tertiary care centres ((9)), but different from the United States Centers for Disease Control and Prevention, which reported pneumonia and CDI as predominant ((7)). While BSI were the most common infection caused by AROs in our study, they were the third least common selected infection overall. Bloodstream infections were also less common than other infections in the United States and in larger Canadian tertiary centres ((7,9)).

Methicillin-resistant *Staphylococcus aureus* was the most common ARO reported in our study, with an infection prevalence of 0.5 per 100 inpatients. This was similar to the MRSA point prevalence reported in 2010, 2012 and 2016 by IPAC Canada point prevalence studies in large hospitals ((5)). Our study revealed a low ESBL-producing organism infection prevalence of 0.4 per 100 inpatients, which is identical to the mean ESBL prevalence reported by IPAC Canada point prevalence studies in 2012 and 2016 ((5)). While the prevalence of ESBLs was low in our study, ESBLs remain an important multi-resistant pathogen in hospitals ((12)) as they are associated with poor patient outcomes, reduced rates of clinical response, longer hospital stays and greater expenses ((13)). This was followed by VRE, with a prevalence of 0.1 infections per 100 inpatients. No patient in our study was infected with CPO. This is consistent with surveillance data that demonstrated that CPO remained infrequently identified in Canadian hospitals ((14)). This may indicate that enhanced infection prevention and control methods can still be used to prevent CPOs from being a common healthcare-associated threat in Canada.

The prevalence of AMU in our study was 35.2%, which was slightly lower than what has been reported from larger Canadian hospitals (39.6% [95% CI, 38.7−40.6] in 2017) ((15)). These surveys reported that the overall prevalence of AMU increased between 2002 and 2009 and stabilized between 2009 and 2017. The prevalence of AMU observed in our study could be due to our patient population. It is possible that patients in smaller community hospitals may have been less acutely ill than those in larger tertiary care centres and therefore required less treatment. Penicillins were the most common drug class prescribed in our study, followed by third-generation cephalosporins, fluoroquinolones, first-generation cephalosporins and carbapenems. This distribution is similar to the distribution of AMU reported from Canadian point prevalence studies ((15)). There is the potential to improve antimicrobial stewardship programs in smaller facilities given that 60.8% of AMU in our study was prescribed empirically. Potential drivers of the decline/stabilization of AMU that has been observed in larger Canadian hospitals could include the development of antimicrobial stewardship programs, changes to antimicrobial prescribing guidelines and changes in patient populations not captured through current survey methods ((15)). These same factors can also impact smaller facilities, including those in our study. Our study used bed size as a proxy for hospital size; however, it should be noted that there is no universal or Canadian definition of a small or large hospital. Despite this, the results from our study of smaller hospitals were similar to what has been observed among larger tertiary care centres.

Screening was conducted to identify clients/patients/residents who were colonized and/or infected with specific AROs. The utility of screening and additional precautions must be weighed against the associated increased healthcare costs, morbidity and mortality of the infection. While it is not a control measure on its own, screening is necessary to apply further infection control measures such as placement and precautions ((16)). In our study, 500 patients (13.7%) were on additional IPAC precautions, and of those, 11.5% were on additional precautions for reasons other than the selected infection types that were under surveillance. Infection prevention and control Canada reported in 2019 that targeted screening was associated with lower rates of MRSA infection ((6)), and all hospitals in our study screened for MRSA on admission and most also screened during the patient’s stay. Our study also demonstrated that 9.1% of hospitals screened for ESBL at admission and 16.4% of hospitals screened during a patient’s stay. This is consistent with prior observations that only a minority of hospitals perform active screening for ESBLs ((12)), as there is a lack of consensus about the value of screening cultures for resistant gram negative bacilli (such as ESBL-producing bacteria) ((16)). The majority (69%) of hospitals screened for CPO and no infections were identified, which may indicate that current levels of IPAC activities are effective. It could also indicate that those infected with CPO are less likely to be in a smaller community hospital and more likely to be at a larger tertiary care centre. Despite an overall increase in VRE infections in Canada ((17)) not all hospitals are screening for VRE at admission ((5,18)), although 71% of the hospitals in our study did so. It is unclear whether all individuals or only high-risk individuals (e.g. surgical patients, intensive care unit patients, patients with a history of colonization) derive more benefit from screening ((18)). Further, other studies have shown that relaxation of some screening protocols may not lead to increasing infection incidence in a hospital setting, advocating that cost effectiveness exercises, with targeted screening and isolation precautions, are crucial ((18,19)).

## Limitations

The main limitation of this study is that prevalence on a single day does not enable a complete understanding of an ARO’s burden and may not be reflective of AMR and AMU time-series trends for each hospital. Furthermore, aggregate infection rates, such as that for pneumonia, may be affected due to seasonal variation. As this study was conducted prior to the coronavirus 2019 pandemic, it is unknown how the changes associated with the pandemic may impact the generalizability of our results. Another limitation of the study is that hospitals were recruited to participate in this study using a convenience sampling method, which can sometimes result in an unrepresentative sample; for example, there was a lack of participation from hospitals located in Canada’s three territories. These hospitals may differ from the hospitals that participated in the survey in important ways, thus impacting the generalizability of our results to facilities in those regions. We recommend that future point prevalence studies improve methodologies and recruitment to align with international standards to enhance national representation and international comparability.

## Conclusion

These data provide information on the prevalence of resistant infections caused by MRSA, VRE, ESBL-producing organisms and CPOs, as well as CDI, among adult inpatients in smaller, northern and rural Canadian hospitals, and complement information published by a Canadian network of larger tertiary care centres ((20)). The findings point to the need for continued study of antimicrobial-resistant pathogens in all Canadian healthcare settings, as rural and community hospitals may represent an important reservoir of AROs.

## Supplemental material

These documents can be accessed on the Supplemental material file.
S1: Hospital survey questions
S2: Patient questionnaire

## Appendix

Appendix A1: Definitions relating to selected infections

Appendix A2: Case definitions relating to selected antimicrobial-resistant organisms

### Appendix A1: Definitions relating to selected infections

An infection is considered to be present if a patient is symptomatic or receiving antimicrobial therapy for the treatment of an infection at the time of the hospital census. Isolates recovered by 8:00 a.m. on the date of the census are eligible for the prevalence survey; please allow one week for laboratory follow-up prior to data submission.

Urinary tract infection (UTI)

Patient must meet Criteria 1a and Criteria 1b:

Criteria 1a

The patient has at least one of the following signs/symptoms:

• Fever >38°C (applicable to patients ≤65 years without an indwelling catheter)

• Suprapubic tenderness with no other recognized cause

• Costovertebral angle pain or tenderness with no other recognized cause

• Urinary urgency (applicable to patients without an indwelling catheter)

• Urinary frequency (applicable to patients without an indwelling catheter)

• Dysuria with no other recognized cause

Criteria 1b

• Positive urine culture ≥10^5^ CFU/ml with no more than two species of microorganisms identified

Skin and soft tissue infection (SSTI)

Patient must meet Criteria 1a and Criteria 1b:

Criteria 1a

The patient has at least one of the following signs/symptoms:

• Patient has purulent drainage, pustules, vesicles, or boils

• Patient has at least two of the following signs or symptoms with no other recognized cause: pain or tenderness, localized swelling, redness, or heat

Criteria 1b

The patient has at least one of the following:

• Organisms cultured from aspirate or drainage from affected site. Note that normal skin flora must be a pure culture. This includes: Diphtheroids, *Corynebacterium* spp., *Bacillus* spp., *Propionibacterium* spp., coagulase-negative staphylococci, (including *S. epidermidis*), viridans group streptococci, *Aerococcus* spp., and *Micrococcus* spp.

• Organisms cultured from blood

• Positive laboratory test performed on infected tissue or blood (e.g. antigen tests for herpes simplex, varicella zoster, *Haemophilus influenzae*, or *Neisseria meningitidis*)

• Multinucleated giant cells seen on microscopic examination of affected tissue

• Diagnostic single antibody titer (IgM) or four-fold increase in paired sera (IgG) for pathogen

Bloodstream infection (BSI)

Patient must meet Criteria 1; or meet Criteria 2a, Criteria 2b, and Criteria 2c.

Criteria 1

• Recognized pathogen cultured from at least one blood culture, unrelated to infection at another site

Criteria 2a

The patient has at least one of the following:

• Fever >38° (core)

• Chills (applicable to patients aged ≥1 year)

• Hypotension

Criteria 2b

• A common skin contaminant cultured from ≥2 blood cultures drawn on separate occasions. This includes: Diphtheroids, *Corynebacterium* spp., *Bacillus* spp., *Propionibacterium* spp., coagulase-negative staphylococci, (including *S. epidermidis*), viridans group streptococci, *Aerococcus* spp., and *Micrococcus* spp.

Criteria 2c

• Positive laboratory results are unrelated to infection at another site

Surgical site infection (SSI)

Patient must meet Criteria 1a and Criteria 1b.

Criteria 1a

The patient has at least one of the following:

• Surgical procedure in the past 30 days

• Surgical procedure in the past 90 days and had an implantable foreign device permanently placed during the surgery

Criteria 1b

The patient has at least one of the following:

• Purulent drainage from superficial or deep incision

• Organism identified from an aseptically obtained specimen from the superficial incision or subcutaneous tissue by a culture or non-culture based microbiologic testing method which is performed for the purposes of clinical diagnosis/treatment

• At least one of the following pain or tenderness, localized swelling, redness, or heat and incision deliberately opened by surgeon/attending physician and non-culture based testing is not performed Surgeon/attending physician diagnoses

• Spontaneous dehiscence or incision deliberately opened or aspirated by a surgeon/attending physician and organism is identified by a culture or non-culture based method which is performed for purposes of clinical diagnosis and treatment and at least one of the following: fever (>38°), localized pain or tenderness

• Abscess/other evidence of infection involving a deep incision found on gross anatomical oar histopathological examination or imaging test

• Infection involves any part of the anatomy deeper than the fascial/muscle layers that was opened/manipulated during operation and at least one of the following: purulent drainage from a drain placed into organ/space, organisms identified from an aseptically obtained fluid or tissue in the organ/space by a culture or non-culture based microbiologic testing method which is performed for the purposes of clinical diagnosis or treatment, abscess/infection involving organ/space found on gross anatomical or histopathological exam, or imaging test suggestive of infection

Pneumonia (PNEU)

Patient must meet Criteria 1a, Criteria 1b, Criteria 1c, and Criteria 1d. Note that patients without underlying pulmonary or cardiac disease (e.g. respiratory distress syndrome, bronchopulmonary dysplasia, pulmonary edema, or chronic obstructive pulmonary disease), one definitive imaging test is acceptable.

Criteria 1a

• Fever >38°

Criteria 1b

• Leukopenia (≤4,00 WBC/mm^3^) or leukocytosis (≥12,000 WBC/mm^3^)

Criteria 1c

Two or more serial chest imaging test results with at least one of the following:

• Infiltrate

• Consolidation

• Cavitation

Criteria 1d

For adults ≥70 years, altered mental status with no other recognized cause, and at least one of the following:

• New onset of purulent sputum or change in character of sputum, or increased respiratory secretions, or increased suctioning requirements

• New onset or worsening cough, or dyspnea, or tachypnea

• Rales or bronchial breath sounds

• Worsening gas exchange (e.g. O_2_ desaturations, PaO_2_/FiO_2_ ≤240), increased oxygen requirements, or increased ventilator demand)

*Clostridioides* (formally *Clostridium*) *difficile* infection (CDI)

Patient must meet Criteria 1a and Criteria 1b; or Criteria 2; or Criteria 3. Note that diarrhea is defined as one of the following: any patient with six or more watery/unformed stools in a 36-hour period; or an adult patient with three or more watery/unformed stools in a 24-hour period that is new or unusual for the patient.

Criteria 1a

• Diarrhea or fever, abdominal pain and/or ileus

Criteria 1b

• Laboratory confirmation of a positive toxin assay or positive polymerase chain reaction for *C. difficile* (without reasonable evidence of another cause of diarrhea)

Criteria 2

• Diagnosis of pseudomembranes on sigmoidoscopy or colonoscopy (or after colectomy) or histological/pathological diagnosis of CDI

Criteria 3

• A diagnosis of toxic megacolon (adult patients only)

### Appendix A2: Case definitions relating to selected antimicrobial-resistant organisms

Methicillin-resistant *Staphylococcus aureus* (MRSA)

• Isolation of *Staphycococcus aureus* from any site

• Resistance of isolate to oxacillin

Vancomycin-resistant *Enterococci* (VRE)

• Isolation of *Enterococcus faecalis* or *faecium*

• Resistance of isolate to vancomycin (minimum inhibitory concentration, MIC ≥8 ug/m)

Extended-spectrum beta-lactamase-producing bacteria (ESBL)

Definitions are given for *Escherichia coli* and *Klebsiella pneumoniae.* Additional ESBLs are to be defined by the reporting facility and indicated as an ESBL on the patient form.

• Isolation of *Escherichia coli* or *Klebsiella pneumoniae* from any site

• MIC testing: a decrease of >3 doubling dilutions in an MIC for either cefotaxime or ceftazidime tested in combination with 4 µg/ml clavulanic acid, versus its MIC when tested alone

• Disk diffusion testing: a >5 mm increase in a zone diameter for either antimicrobial agent tested in combination with clavulanic acid versus its zone when tested alone

Carbapenemase-producing organisms (CPOs): *Enterobacteriaceae* spp. and *Acinetobacter* spp.

All *Enterobacteriaceae* spp. and *Acinetobacter* spp. that demonstrate resistance to carbapenem-class antimicrobials (defined below) should be investigated for the production of carbapenemase.

Carbapenem-resistance is defined as:

• *Enterobacteriaceae* carbapenem-resistant organism (CRO):

o Imipenem, meropenem, or doripenem resistance: (MIC ≥2 μg/ml) or (≤22 mm disk diffusion)

o Ertapenem resistance: (MIC ≥1 μg/ml) or (≤21 mm disk diffusion)

• *Acinetobacter* CRO:

o Imipenem or meropenem resistance: (MIC ≥8 μg/ml) or (≤15 mm disk diffusion)

Carbapenemase-producing organism (CPO):

• Organisms (e.g. *Enterobacteriaceae* spp. and *Acinetobacter* spp.) identified as a CPO must meet hospital or provincial definitions. CPOs do not need to meet the CRO definitions, above, and supersede CRO status if applicable
